# Buffalo Medical College

**Published:** 1848-01

**Authors:** 


*Buffalo Medical College.—Extension of Lecture Term.*—The Faculty of this institution have resolved that the lecture session for 1848-9 shall commence on the last Wednesday in Nov., 1848, making, with the preliminary dissecting term of one month, a *six months course*, thus, virtually, if not literally, adopting the recommendation of the National Medical Convention.

*The coming Spring Course, commencing the last of February next, will continue, as announced in the Circular and advertisements, for the usual period, viz. sixteen weeks.*

We take great pleasure in making this announcement.

The Buffalo Institution is, so far as we know, the fourth Institution in the country to extend the lecture term, and the first to do so of the Institutions north and west of New York city,

